# Downregulation of MiR-31 stimulates expression of LATS2 via the hippo pathway and promotes epithelial-mesenchymal transition in esophageal squamous cell carcinoma

**DOI:** 10.1186/s13046-017-0622-1

**Published:** 2017-11-16

**Authors:** Yanping Gao, Jun Yi, Kai Zhang, Fan Bai, Bing Feng, Rui Wang, Xiaoyuan Chu, Longbang Chen, Haizhu Song

**Affiliations:** 1 Department of Medical Oncology, Jinling Hospital, Medical School of Nanjing University, 305 Zhongshan East Road, Nanjing, Jiangsu 210002 China; 2 Department of Cardiothoracic Surgery, Jinling Hospital, Medical School of Nanjing University, 305 Zhongshan East Road, Nanjing, Jiangsu 210002 China; 3 Department of Medical Oncology, Nanjing Clinical Medical School of the Second Military Medical University, Nanjing General Hospital of Nanjing Military Command, PLA, Nanjing, 210002 China

**Keywords:** miR-31, LATS2, Hippo pathway, TAZ, EMT, Esophageal squamous cell carcinoma

## Abstract

**Background:**

Dysregulation of miRNAs is associated with cancer development by coordinately suppressing abundant target genes. Emerging evidence indicates that miR-31 plays a dual role in tumorigenicity. However, whether miR-31 plays as an oncogene in esophageal squamous cell carcinoma (ESCC) and the potential target molecules are still unclear. MiR-31 role in ESCC was investigated and an association of the target molecules with EMT was identified in the progression of ESCC.

**Methods:**

Western blot assays and qRT-PCR was performed to detect the protein and mRNA levels. We investigated the role of miR-31 in the regulation of LATS2 expression in ESCC cell lines via functional assays both in vivo and in vitro. The luciferase reporter assays was conducted to confirm LATS2 is a potential target of miR-31. Immunohistochemistry was used to measure LATS2 and TAZ expression in normal and ESCC tissue.

**Results:**

LATS2 is a component of the Hippo tumor-suppressive signaling pathway. Frequent loss of heterozygosity of LATS2 has been reported in esophageal cancer. We analyzed the reciprocal expression regulation of miR-31 and LATS2 and demonstrated that LATS2 expression was elevated by down-regulation of miR-31 at the post-transcriptional level in ESCC. Moreover, miR-31 significantly suppressed the luciferase activity of mRNA combined with the LATS2 3′-UTR, a key molecule in the Hippo pathway. Then, LATS2 consequently promoted the translocation of TAZ, which was examined using immunohistochemistry. Silencing of miR-31 significantly inhibited the cell proliferation, induced apoptosis and decreased the ability of migration/invasion in vitro. LATS2 impedes ESCC cell proliferation and invasion by suppressing miR-31, as well as mice xenograft model in vivo. Meanwhile, the nuclear localization of LATS2 constrained the phosphorylation of TAZ. Then, the expression level of TAZ was notably heightened with a high risk of recurrence compared to that observed in the low-risk patients, as well as, the higher expression associated with a poor survival.

**Conclusions:**

Our study demonstrated that overexpression of miR-31 undertook an oncogenic role in ESCC by repressing expression of LATS2 via the Hippo Pathway and activating epithelial-mesenchymal transition. LATS2 and TAZ could be potential novel molecular markers for predicting the risk of recurrence and prognosis of ESCC.

**Electronic supplementary material:**

The online version of this article (10.1186/s13046-017-0622-1) contains supplementary material, which is available to authorized users.

## Background

Esophageal cancer is one of the most widespread types of malignant tumor, which is the sixth leading cause of cancer-related deaths in the world and third in China [1, 2]. Esophageal squamous cell carcinoma (ESCC), the predominant histologic subtype of esophagus cancer, is prevalent in Asia, accounting for 90% cases especially in China [3–5]. Due to a spectrum of aberrantly aggressive phenotypes and lack of early detection, most of the patients are diagnosed with advanced disease and have to give up the main curative option of surgical resection. Despite recent advances in multimodality therapies, the prognosis remains dismal. Like other malicious tumors, the pathogenesis and progression of ESCC are a long procedure involving activation of oncogenes and/or inactivation of tumor suppressor genes. Recently, promising molecular genetic alterations with clinical outcome in ESCC have been predicted [6, 7]. Therefore, specific molecular markers associated with the progression and therapeutic targets are immediately needed for patient classification and the improvement of individualized therapy regimens.

MicroRNAs (miRNAs) are a class of highly-conserved, non-coding RNAs of 18 to 25 nucleotides in length and could function as indispensable and negative regulators of gene expression at the post-transcription level. The mature forms of miRNAs silence the gene expression by binding to the 3′-untranslated region (3′-UTR) of mRNAs and initiate the translational repression and/or target them for degradation. Mounting evidences indicate that miRNAs can donate to the malignant tumor progression and metastasis process, such as cell proliferation, invasion, angiogenesis, and the epithelial to mesenchymal transition (EMT) [8–10]. Among the most frequently altered miRNAs identified, miR-31, which is located on the common homozygous deletion region on chromosome 9p21.3, is emerging as a complex player in an ocean of cancers. Evidence proposes that miR-31 can function as either an oncogene or a tumor suppressor in type-specific cancers, respectively. For example, increased expression of miR-31 has been identified in colorectal [11], lung cancer [12]and HNSCC [13], whereas it plays a tumor-suppressive role in ovarian [14] prostate [15], breast cancer [16] and melanoma [17]. Moreover, downregulation of miR-31 in esophageal adenocarcinoma (EAC) correlates with poor prognosis [18, 19]. Inversely, miR-31 is up-regulated in tissue and serum samples of ESCC, with expression relating to staging [20]. Still, in another ESCC miR-31 expression was diminished [21]. These studies emphasize the complexity of miR-31-associated malignant phenotypes. Challenges have to be resolved before miR-31 could be investigated in clinical trials, including definition of miR-31 targets, as well as pathways regulating miR-31 expression in ESCC.

The Hippo pathway is an evolutionarily conserved pathway that exerts profound effects on the regulation of organ size, tumorigenesis, embryonic development, stem cell homeostasis, and epithelial to mesenchymal transition [22]. One of the cores of Hippo signaling complex in mammals is Lats1 or Lats2 (Lats1/2) kinases, others including MST1/2, MOB1 and YAP1 [23, 24]. LATS2 kinases are members of the LATS/NDR kinase family, which encodes a serine/threonine protein kinase belonging to a sub-group of AGC (protein kinase A (PKA)/PKG/PKC-like) kinases [25]. LATS2 gene has been located onto chromosome 13q11–12, a hot spot region as a tumor suppressor [26]. LATS2 acts a meaningful role in centrosome duplication and maintenance of mitotic fidelity, because its protein localizes to centrosomes during interphase as well as early and late metaphases [27]. LATS2 can inhibit cell growth at the G1/S transition via downregulating cyclin E/CDK2 kinase activity [28], and induction of apoptosis via down-regulation of apoptosis inhibitors such as Bcl-2 and Bcl-xL [29]. Once Hippo is activated, MST1/2 phosphorylates LATS1/2. Then the activated Lats1/2, in association with the tumor suppressor Mob1, in turn phosphorylates and inactivates transcriptional coactivators TAZ and YAP by their cytoplasmic retention and proteasome-mediated degradation [30]. However, TAZ and YAP can be recruited to their target promoters through binding to the TEAD/TEF transcription factors instead of directly binding to DNA [31] where they control the transcription of genes critical for EMT, cell proliferation, apoptosis, survival, differentiation, and cancer stem cell expansion [32–35]. The activity of the Hippo pathway, especially TAZ/YAP, can be regulated by growth factors and extracellular diffusible signals as well as signals generated through cell-cell junction, tissue architecture, and mechanotransduction [36]. It was also shown that dysregulation of the Hippo pathway is associated with epithelial-mesenchymal transition and cancer development, mainly driven by TAZ and YAP [37]. Obviously, a bidirectional relationship exists between EMT and TAZ/YAP, whereby the loss of polarity and cell contacts stimulates the activation of both factors, which in turn participate in the EMT program [38]. Furthermore, Muramatsu T et al. has demonstrated that YAP was frequently overexpressed in ESCC and they also showed that patients with YAP-overexpressing tumors had a worse overall rate of survival than those with non-expressing tumors. Their results have ultimately indicated that YAP is a putative oncogene in ESCC and it represents a potential diagnostic and therapeutic target [39]. Thus we tended to focus on exploring the roles of TAZ in the ESCC epithelial-mesenchymal transition and chemoresistance. To date, numerous miRNAs have been verified to target LATS2 and involved in Hippo pathway in diverse types of cancer, like miR-181b, miR-93, and miR-372 [40–42]. However, the specific expression features of miR-31 in ESCC remains undefined, and the underlying mechanisms of miR-31/LATS2 axis regulating epithelial-mesenchymal transition are still unknown.

Herein, the role of LATS2 and TAZ in miR-31 repression and the contribution of miR-31 to proliferation, migration, invasion and EMT of ESCC was explored. We identified that miR-31 directly suppressed LATS2 expression, which inactivated TAZ and led to the subsequent action of ESCC tumorigenicity. Significantly, it was showed that LATS2 and its downstream gene TAZ highly correlated with ESCC progression with poor prognosis. Altogether, these results suggested that miR-31 might act as a biomarker in ESCC and a novel functional axis of miR-31/LATS2/TAZ might propose a feasible therapeutic approach for ESCC that merited further evaluation.

## Methods

### Cell culture

The human normal esophageal epithelial cell line HEEC and human ESCC cell lines (Kyse30, Kyse70, Eca109, Ec9706 and TE1) were purchased from Tumor Cell Bank of the Chinese Academy of Medical Science (Shanghai, China). HEEC, Kyse30, Kyse70, TE1 and Eca109 cells were maintained in RPMI-1640 medium (Gibco, USA) containing 10% fetal bovine serum (FBS, Gibco, USA) and 1% penicillin/ streptomycin (Invitrogen, Shanghai, China). Ec9706 cells were expanded in DMEM medium (Gibco, USA) supplemented with 10% FBS and 1% penicillin/streptomycin. Cells were all cultured at 37 °C in a humidified atmosphere of 95% air and 5% CO_2_.

### Microarray array analysis

Total RNA was extracted from five pairs of ESCC tumor and adjacent normal tissues using the mirVana miRNA isolation kit (Ambion, USA). Microarray chip analysis was performed and analyzed by Exiqon (Vedbaek, Denmark). The fold-change was calculated by comparing the expression level of miRNAs in the ESCC tumor pool and with that of the normal tissue pool using a log_2_ format.

### MiRNA target prediction

Five established miRNA-target prediction programs (TargetScan, miRanda, PicTar, MirTarget2, and PITA) were employed to predict miRNA targets, with genes predicted by all five independent tools considered. The selected genes of each individual miRNA were subjected to GO and pathway analysis.

### Plasmid construction and cell transfection of oligonucleotides and plasmids

We selected Eca109 and TE1 cells for further functional research. MiR-31 mimic, inhibitor and their corresponding controls were purchased from realgene biotechology (Nanjing, china). Has-miR-31 mimic:AGGCAAGAUGCUGGCAUAGCU, CUAUGCCAGCAUCUUGCCUUU; mimics NC:UUCUCCGAACGUGUCACG UTT,ACGUGACACGUUCGGAGAATT; Has-miR-31 inhibitor: AGCUAUGCCAG CAUCUUGCCU; Inhibitor NC:CAGUACUUUUGUGUAGUACAA. For the reduction and induction of LATS2 expression, cDNA/pLATS2, siRNA/LATS2 and matched controls (cDNA/pNC and siRNA/NC) plasmids were purchased from Shanghai GenePharma Co.,Ltd. Online design software was applied to design primers and BLAST homology screening was subsequently performed. Primers sequences as follows: sense: CTTCTATTATAAAATTACCATATATTATTATTCACAGC AGGTCCTGTGAATAC, reverse: CTTCTATTATAAAATTACCATATATTATTATTCACAGCAGGTCCTGTG AATAC. Cells were planted into 6-well plates (2 × 10^5^ cells/well) and transfected with 100 pmol miRNAs or 4μg DNA 4μg DNA according to the manufacturer’s protocol. Stably-transfected cells were selected for 14 days in the presence of 2 μg/mL puromycin (Sigma, USA).

### RNA extraction and qRT-PCR analysis

Total RNA was extracted from surgical tissue specimens and the cultured cells using Trizol reagent (Invitrogen, CA, USA) according to the manufacturer’s protocol and concentration was measured by a spectrophotometer. For reverse transcription, cDNA was conducted using TaqmanTM microRNA reverse transcription kit and performed to real-time PCR using TaqManTM MicroRNA Assay kit (Applied Biosystems, USA) based on the manufacturer’s instructions. Relative quantification was achieved by normalization to the amount of GAPDH mRNA. The primers for miR-31 were F: 5′-CAGCTATGCCAGCATCTTGCCT-3′. About U6, the primers were F: 5-GCGCGTCGTGAAGCGTTC-3, R: 5-GTGCAGGGTCCGAGGT-3. The primers of LATS2 were F: ATGAGCTCCACTCTGCTCAATGTCACGG, R: GCAAGCTTCTCTA CCAAGAATGAAAGAGCAT. The primers for GAPDH were 5′-GCACCGTCAAG GCTGAGAAC-3′ and 5′-TGGTGAAGACGCCAGTGGA-3′. The primers for TAZ were F: GAATTCATGAATCCGGCCTCGGCGCCCC, R: GGATCCTTACA GCCACCTTAGAAAGGGC. The primers for E-cadherin were F: TTGTGGCAGAGTGTAATGCTG, R:GTCCCTGGTCTTCTTGGTCA;B-catenin were F: GCTGGTGACAGGGAAGACAT, R: CCATAGTGAAGGCGAACTGC; N-cadherin were F: CAAACAAGGTGAG ACGATGC,R: GCCAGGATGAGTAAGCG TGT; Vimentin were F: AGAGAACTTTGCCGTTGAAGC, R: ACGAAGGTGACGAGCCATT. Relative gene expression levels were calculated by the ΔΔCt method. All reactions were performed in triplicate.

### Western blot analysis

Cells were harvested directly or 48–72 h after transfection. Cells and tissues were lysed with ice-cold RIPA buffer supplement with Phenylmethanesulfonyl Fluoride (PMSF) and cocktail. Cell protein lysates were subjected in 10% sodium dodecyl sulfate-polyacrylamide gels, electrophoretically transferred to polyvinylidene difluoride membranes (Roche). Protein loading was estimated using mouse anti-GAPDH monoclonal antibody. The membrane was incubated with 5% skim milk, washed and then incubated with the rabbit anti-human LATS2(1:2000 dilution)and TAZ (1: 1000 dilution) and GAPDH (1: 5000 dilution) overnight at 4 °C, followed by blotted with secondary antibody conjugated to horseradish peroxidase for 1 h at 37 °C. All antibodies were purchased from Abcam (Abcam, USA). The proteins were perceived by the enhanced chemiluminescence kit (Invitrogen) and exposed to x-ray film. Protein levels were normalized to GAPDH.

### Methylthiazolyldiphenyl-tetrazolium bromide (MTT) survival assay

Cell proliferation was analyzed using MTT assay. In total, 5 × 10^3^ transfected cells were seeded into each well of a 96-well plate in a final volume of 100 mL and cultured with the desired drug or drug combination for 24–72 h. Next, 20uL MTT solution (5 mg/mL) was added to the cells for 4 h at 37 °C. After removing the medium, the remaining MTT formazan crystals were solubilized in dimethyl sulfoxide (DMSO). The relative number of surviving cells in each group was measured by using a microplate reader (Bio-Rad, Model 680) at 560 nm.

### Colony formation assay

Cells were cultured to single cell suspensions and seeded into 6-well plates in triplicate (500 cells/well) for approximately 24 h under standard conditions. With specific treatments directly or 48 h after transfection, and cells were allowed to grow for 10–14 days. To visualize colonies, cells were fixed with methanol and stained with 0.5% crystal violet. Colonies with ≥50 cells were visible colonies, which were manually calculated.

### Wound healing assay

The wound healing assay was performed to assess cell migration ability. Transfected Eca109 and TE-1 cells and their NC were seeded onto a 6-well culture plate (5 × 10^5^) and cultured to a subconfluent state in complete medium. After 24-h starvation in serum-free medium, an artificial wound was linearly scraped on the confluent cell monolayer using a standard P-200 pipette tip. Cells that had detached from the bottom of the wells were gently aspirated. Then cells migrated into the scratch area as single cells from the confluent sides. The width of the scratch gap was monitored under an inverted microscope and photographed at 0 h and 48 h. In terms of the difference between the original width of the wound and the width after cell migration was quantified. Three replicates of each condition were used.

### Transwell migration and invasion assays

For the Transwell migration assay, the above-transfected cells were plated to the upper chambers of 8-μm pore polyethylene membranes Transwell plates (Corning, MA, USA) and each insert that had not been coated with Matrigel (BD Biosciences, San Jose, CA, USA). For the Matrigel-coated Transwell invasion assay, pre-coated Matrigel and transfected cells were placed in the upper chambers of Transwell plates. All experiments were performed at least three times in triplicate. Cells (5 × 10^4^ for the migration assay; 1 × 10^5^ for the invasion assay) were added to the upper chamber with serum-free medium, and the lower chamber contained culture medium with 20% FBS to act as a chemoattractant. Approximately 24 h after seeding at 37 °C in 5% CO_2_, cells that appeared on the undersurface of the filter were fixed with methanol, stained with 0.1% crystal violet and cells on the undersides of the filters were observed and counted under a microscope. All experiments were executed at least three times in triplicate.

### Immunohistochemistry

According to the routine protocol, primary ESCC tissues and adjacent normal tissues attained after surgery were performed to IHC analysis. Firstly, formalin-fixed paraffin-embedded tissue specimens were sectioned into 3 μm thickness for immunohistochemistry. Then for deparaffinage, the samples sections were treated with xylene, and hydrated through a sequence of decreasing concentrations of ethanol to water. For high-temperature antigen retrieval, slides were incubated with citrate buffer solution (Maixin Bio, China) at 100 °C for 1 min. Next, slides were immersed in 100 μl of 3% hydrogen peroxide for 10 min at room temperature to block endogenous peroxidase activity. After washing with phosphate-buffered saline (PBS) 3 times, the sections were incubated with 5% bovine serum albumin (BSA; Sigma-Aldrich, USA) for 30 min, followed by incubation with a monoclonal mouse anti-LATS2 antibody (1:250, Abcam, UK) and anti-TAZ antibody (1:100, Abcam, UK) at 4 °C overnight. After washing with PBS, the sections were incubated with secondary antibody for 30 min at 37 °C. Subsequently, diaminobenzidine was used as the colorizing reagent, and hematoxylin was used to counter stain nuclei. PBS was used as a negative control for the staining reactions. Finally, all sections were dehydrated in increasing concentrations of ethanol and xylene and were mounted with neutral gum.

Stained sections were scored by three pathologists independently to reach interobserver agreement. Each section was scored according to the intensity and percentage of positive cells. That is, staining intensity was scored as follows: 0 (negative), 1 (weakly positive), 2 (moderately positive), and 3 (strongly positive). The percentage of positive cells was also graded according to four categories, 1 point for less than 10% positive cells; 2 for 10–50% positive cells; 3 for 51–80% positive cells; and 4 for more than 80% positive cells. Overall scores ≤6 were defined as low expression, and scores >6 were defined as high expression.

### Immunofluorescence staining

Cells were transplanted on sterilized cover slips cultured at approximately 80% confluence for about 24 h. For immunofluorescence processing, they were fixed in ice-cold acetone for 15 min, washed with PBS twice, and then stained with rabbit anti E- cadherin, N-cadherin, Vimentin and β-catenin overnight at 4 °C after blocking with 3% BSA for 30 min at room temperature. After washing, the cells were incubated with goat anti-rabbit FITC conjugated secondary antibody for 30 min at room temperature. Then Nuclei were counterstained with 4–6-diamidino- 2-phenylindole (DAPI) for 2 min at room temperature. The coverslips were mounted and imaged under a fluorescent microscope. The indirect immunofluorescence analysis was valued and performed by IPWIN60.

### Luciferase reporter assay

The luciferase reporter assays was conducted according to the manufacturer’s instructions (Dual-Glo Luciferase Assay System, Promega cat. no. E2920). The pLUC firefly luciferase vectors contained empty, wild type, and mutant LATS2 3′-UTR sequence, respectively. Luciferase activity assays for miR-31 target validation were performed 48 h after transfection. The relative luciferase activities were normalized by Renilla luciferase activities. Each sample was measured in triplicate, and the experiment was repeated at least three times.

### Mice xenograft models and immunohistochemistry analysis

All animal studies were conducted in accordance with protocols that were approved by the Jiangsu Province Animal Care and Use Committee. In this study, all female BALB/c athymic nude mice at 4–6 weeks of age were purchased from the Department of comparative medicine (Jinling Hospital, Nanjing, China). Exponentially growing cells were split and grown in a fresh medium for one more day before harvest for inoculation. Nearly 5 × 10^6^ Eca109 and TE1 cells were suspended in 100 μL PBS and subcutaneously inoculated on the right side of the posterior flank. Beginning day 6 after injection, tumor diameters were measured every other day. Tumor volume was calculated using the equation: V = A× B^2^/2 (mm^3^), with A being the largest diameter and B being the perpendicular diameter. After 30 days, all mice were sacrificed, and transplanted tumors were excised. The primary tumor tissues were performed H&E staining and TUNEL staining. The ethics committee of Jiangsu Province Medical Association approved the study protocol.

### Patients and tissue samples

A total of 164 primary ESCC tissues specimens were collected from patients at Cardiothoracic Surgery Department of Jinling Hospital (Jiangsu, China). None of the patients with ESCC had received radiotherapy or chemotherapy before surgery. Patients met all of the following criteria: patients who suffered from primary ESCC, a histological diagnosis of ESCC with at least one measurable lesion; a clinical stage of I–III. Diagnosis of ESCC was determined according to the latest All patients enrolled in our study received standard postoperative adjuvant therapy according to the NCCN guidelines for esophageal cancer and pTNM stage classification (AJCC). Patient tissue samples were fixed by the fixed, paraffin embedded. Clinical and pathologic characteristics, including age, gender, operation time, tumor stage, and related pathologic data, were collected retrospectively from the patient records (Tables 2 and 3). Written informed consent was obtained from all patients in the study. The research protocol was reviewed and approved by the Ethical Committee and Institutional Review Board of the Jinling Hospital.

### Statistical analysis

Data were expressed as mean ± SEM from at least three independent experiments. Survival time was defined from the date of surgery to the latest follow-up or the date of death. Continuous data were compared between groups using the Mann–Whitney U test. Categorical variables of the clinical specimens examined were compared using chi-square test (or Fisher’s exact test where appropriate). Survival analysis using the Kaplan-Meier method was performed using the log-rank test. The relationship between two variables and numerical values obtained by real-time quantitative RT-PCR were analyzed using Student’s t-tests. Multiple group comparisons were analyzed with one-way ANOVA. In the same ESCC patients, the correlation of miR-31 and LATS2/TAZ and the correlation of LATS2 and TAZ was performed Pearson Correlation analysis. All statistical analyses were performed using SPSS19.0 software (SPSS Inc., USA). *P* < 0.05 was considered statistically significant.

## Results

### MiR-31 is upregulated in ESCC cell lines and tissues

**Fig. 1:**
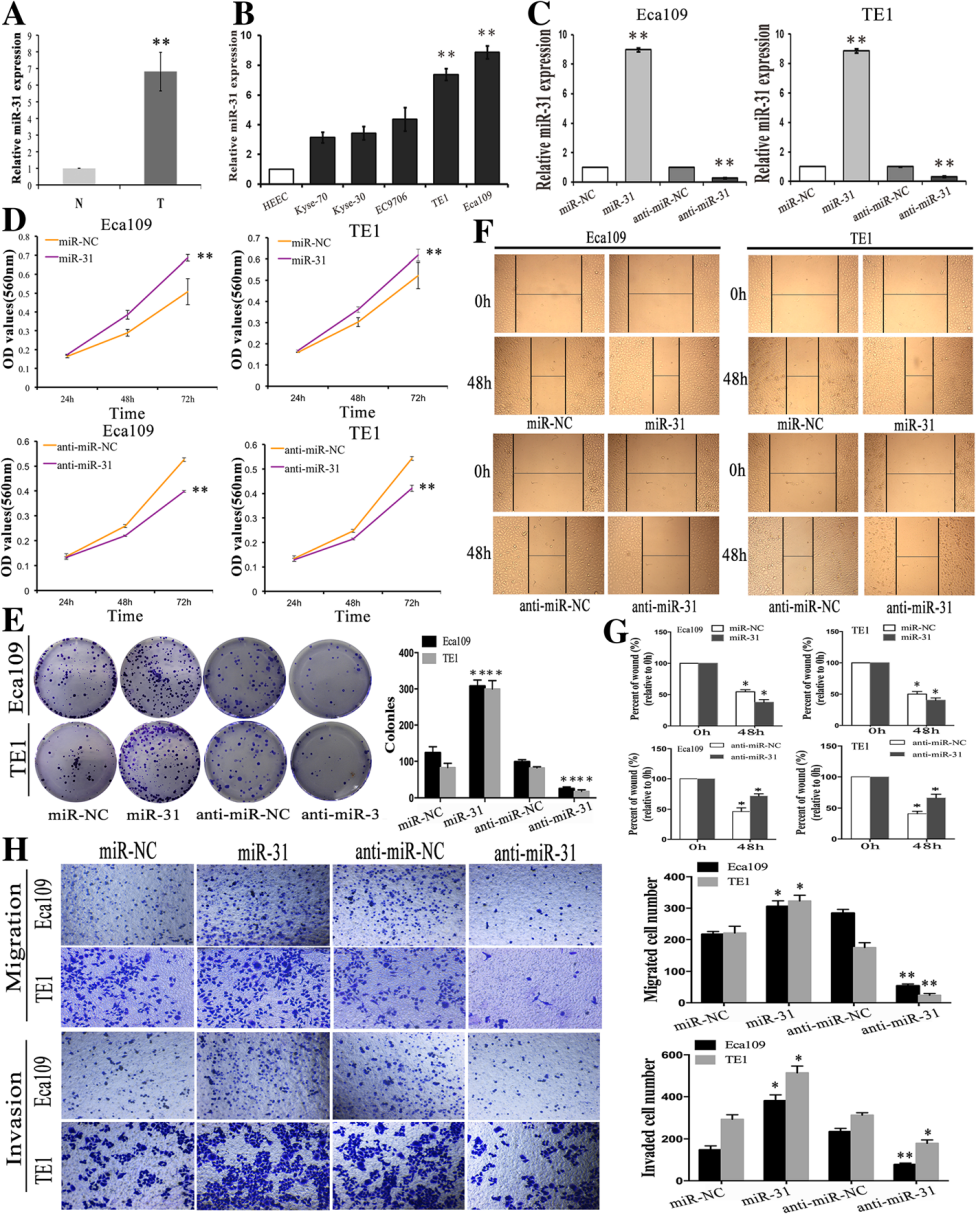
Expression of miR-31 in ESCC cell lines and tissue samples and the in vitro effects of miR-31 on cell proliferation, migration and invasion in ESCC cells. **a** The relative expression level of miR-31 in 20 specimens of ESCC (T) and the adjacent nontumor tissues (N) was determined by qRT-PCR. **b** QRT-PCR analysis miR-31 expression in five human ESCC cell lines and the normal esophageal epithelial cell line (HEEC). **c** QRT-PCR analysis of the relative expression of miR-31 in each group of ESCC cells transfected with miR-31 mimics and inhibitor. **d**-**e** MTT and colony formation assays in ESCC cells overexpressing or underexpressing miR-31. **f**-**g** Wound scratch healing assay of ESCC cell showed that change of miR-31 effectively affected cell motility. Photographs were taken immediately (0 h) and at 48 h after wounding, quantification of wound closure was done. **h** Migration assay and Invasion assay revealed that the upexpression or downexpression of miR-31 promoted or inhibited the invasion ability of ESCC cells. Results are expressed as the mean ± SD of three independent experiments. *: *P* < 0.05; **: *P* < 0.01

As mentioned above, the complexity of miR-31-associated phenotypes and investigations of miR-31 function in ESCC were inadequate. Firstly, comparison of the miRNA expression profiles, determined by microarray analysis between normal esophageal epithelium tissues and esophageal squamous carcinoma tissues, indicated that 2000 human miRNAs showed various degrees of expression. Through global normalization of the raw data, 34 differentially expressed miRNAs were identified in ESCC tumor tissues compared with their squamous epithelial tissues when using a 1.5-fold change cut-off point (Table 1). In the same way, microarray screening showed miR-31 to be upregulated in ESCC when compared to normal epithelia in other study [20]. Then, further exploration in the significance of miR-31, as well as pathways regulating miR-31 expression in ESCC, qRT-PCR analysis of miR-31 expression in 20 human ESCC tissue samples and their matched normal tissues was carried out, also in a normal human esophageal cell line (HEEC) and a panel of ESCC cell lines. These specimens were from three primary ESCC cases treated by the Cardiothoracic Surgery Department of Jinling Hospital. The results showed the expression of miR-31 was significantly up-regulated in ESCC tissues when compared to the adjacent non-tumor tissues, these higher levels were displayed in ESCC cells when compared to HEEC cell lines (Fig. 1a, b). It is not difficult to find miR-31 was up-regulated which was consistent with qRT-PCR results.

**Table 1:** Differentiated dysregulation of miRNAs in ESCC

miRNA	Fold change	
hsa-miR-99a-5p	5.64	down
hsa-miR-140-3p	5.21	down
hsa-miR-125b-5p	5.08	down
hsa-miR-30b-5p	4.99	down
hsa-miR-126-3p	4.63	down
hsa-miR-30a-5p	4.48	down
hsa-miR-26a-5p	4.24	down
hsa-miR-29a-3p	4.01	down
hsa-miR-150-5p	3.43	down
hsa-miR-142-5p	3.22	down
hsa-miR-27b-3p	3.13	down
hsa-let-7a-5p	2.38	down
hsa-miR-22-3p	2.21	down
hsa-miR-6069	2.18	down
hsa-let-7f-5p	2.15	down
hsa-miR-193b-3p	2.14	down
hsa-miR-31-5p*	2.63	up
hsa-miR-1273e	2.48	up
hsa-miR-483-5p	2.45	up
hsa-miR-4793-5p	2.44	up
hsa-miR-6510-5p	2.37	up
hsa-miR-642b-3p	2.15	up
hsa-miR-765	2.11	up
hsa-miR-371a-5p	2.07	up
hsa-miR-4485	2.04	up
hsa-miR-4800-5p	2.02	up
hsa-miR-3141	1.98	up
hsa-miR-575	1.96	up
hsa-miR-21-5p	1.94	up
hsa-miR-93-5p	1.84	up
hsa-miR-4499	1.77	up
hsa-miR-188-5p	1.62	up
hsa-miR-106b-5p	1.54	up

^*^MiRNAs with more than 1.5-fold expression change in ESCC tissues relative to expression in matched normal tissues

### Knockdown of miR-31 depresses proliferation, migration, and invasion in ESCC cells

To further define the potential biological significance of miR-31 in ESCC, we firstly used miR-31 mimics and antagonist to perform the gain and loss function analysis. Effective expression of miR-31 in stable transfectants was confirmed by qRT-PCR (Fig. 1c). Overexpression of miR-31 increased ESCC cells proliferation and cell survival, as shown by MTT assays (Fig. 1d) and colony formation (Fig. 1e). Conversely, downregulation of miR-31 diminished ESCC cells proliferation (Fig. 1d, e). Meanwhile, the effects of miR-31 on migration and invasion of ESCC cells were further investigated. Clearly, wound-healing assay disclosed that overexpression of miR-31 promoted the migratory activity of ESCC cells, yet the migratory ability of ESCC cells stably transfected with anti-miR-31 was significantly lower than that of cells transfected with anti-miR-NC (Fig. 1f, g). Next, we investigated whether cell mobility was affected, by performing an invasion assay finding that knockdown of miR-31 repressed the invasive ability of ESCC cells. Analogously, cell invasion was reduced in anti-miR-31-transfected cells as determined by the matrigel invasion assay, and enforced expression of miR-31 caused the contrary effect (Fig. 1h). These outcomes suggested that anti-miR-31 could effectively inhibit the growth, migration and invasion of ESCC cells in vitro.

### LATS2 was identified as a direct and functional target of miR-31 in ESCC cells

**Fig. 2:**
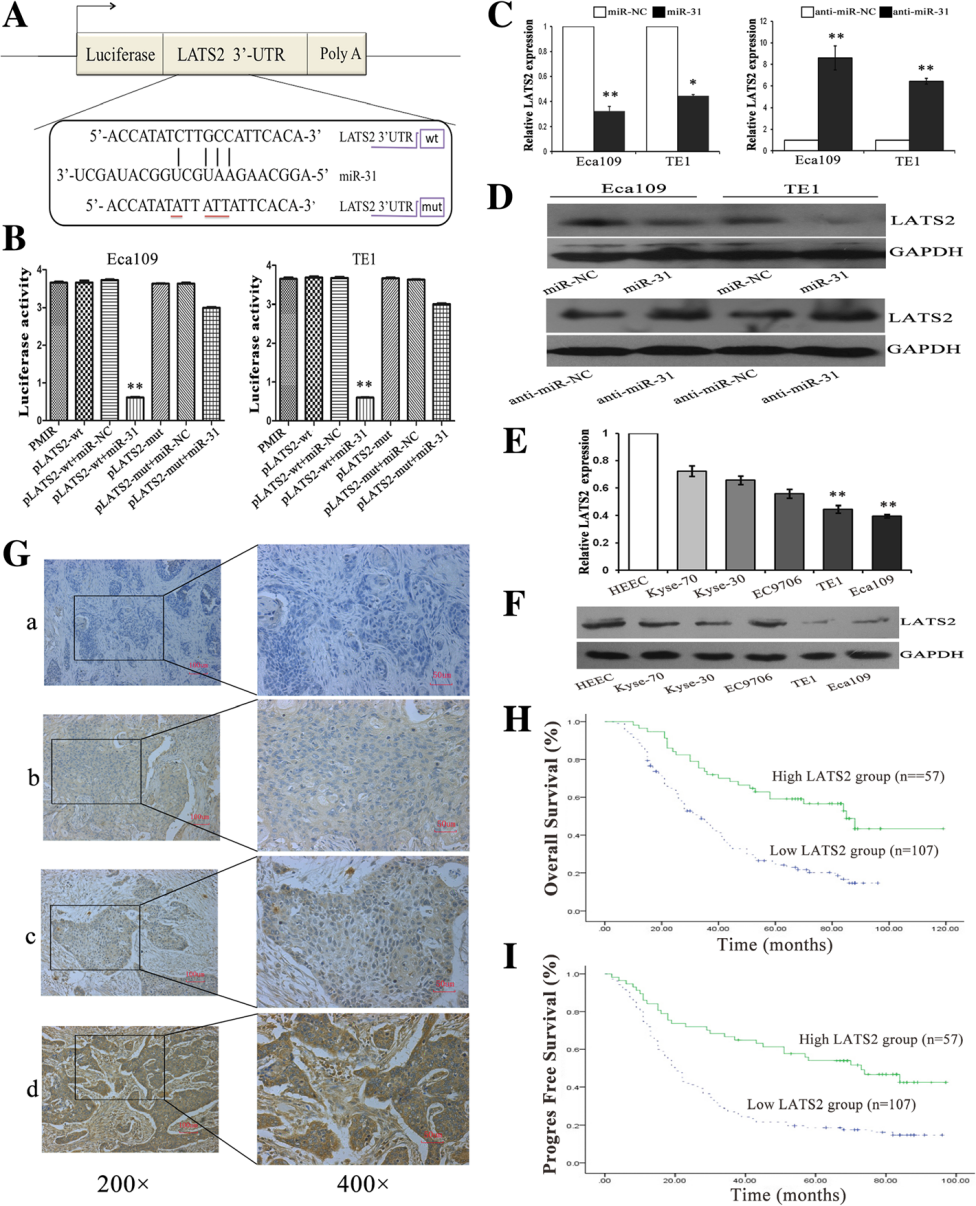
MiR-31 negatively regulates the expression of LATS2 by directly targeting the LATS2 3′-UTR. **a** Schematic representation of miR-31 gene promoter with the putative LATS2-binding sites and the sequences of the point mutations. **b** Luciferase activity in cells following co-transfection with control or miR-31-encoding plasmids and wild or mutant LATS2 pLUC vectors to predict miR-31 binding site in the 3′-UTR of LATS2. **c**-**d**. The mRNA and protein levels of LATS2 were detected in the group of ESCC cell lines than HEEC cell lines via qRT-PCR and western blot assays. **e**-**f**. QRT-PCR and western blot analysis showed that miR-31 could negatively regulate LATS2 expression of ESCC cells. **g**. Immunohistochemical staining of LATS2 protein in primary ESCC tissue samples (Left: ×200; Right: ×400). **h**-**i**. Kaplan-Meier survival plots analysis of the association of LATS2 with the OS and PFS of ESCC patients. Values are mean ± SD; *: *P* < 0.05; **: *P* < 0.01

In order to clarify the mechanisms by which miR-31 promotes tumorigenesis, the target mRNAs of miR-31 was identified in silico prediction models [43]. Among numerous candidates, we focused on LATS2 because it is a recognized tumor suppressor gene, which has been reported to be a direct target of miR-31 in some researches [12, 44, 45]. The decent binding site for miR-31 was actually found in the 3′-UTR region of LATS2 mRNA. To confirm the direct binding and function of miR-31 upon LATS2, both wild and mutated 3’UTR sequences of LATS2 promoter were designed and cloned into the basic firefly luciferase reporters and co-transfected with miR-31 into Eca109 and TE1 cells (Fig. 2a). The detection of a normalized luciferase activity revealed that miR-31 notably inhibited the activity of luciferase combined with wild-type LATS2 3′-UTR (*p* < 0.01). Importantly, when the binding sequences were mutated, the suppressive effects of miR-31 on luciferase activity were attenuated (Fig. 2b), implying the direct negative regulation of miR-31 on the promoter region of LATS2.

To further verify that LATS2 is a target of miR-31 in ESCC cells, we detected the expression of LATS2 in a normal human esophageal cell line (HEEC) and a panel of ESCC cell lines via qRT-PCR and western blot analysis at first. As shown in Fig. 2e and f, the mRNA and protein levels of LATS2 were both significantly low expressed in the group of ESCC cell lines than HEEC cell lines. Then, the mRNA and protein levels of LATS2 were analyzed in Eca109 and TE1 cells after mutable expression of miR-31. We discovered that LATS2 was downregulated in the augment of miR-31, and LATS2 was enhanced after using the miR-31 inhibitor compared with that observed in the control cells (Fig. 2c, d).

### Association of LATS2 expression with clinicopathological characteristics of ESCC patients

To address the clinical significance of LATS2 in ESCC, the samples of 164 patients were used on evaluating correlations between LATS2 expression level and clinicopathological features. To start with, LATS2 were analyzed by MaxVision immunohistochemical method in 164 ESCC tissues. Lats2 was expressed in cytoplasm and staining intensity was scored as follows: a (blank control), b (weak staining), c (moderate staining), and d (strong staining). PBS solution was used as blank control (Fig. 2g). The high expression rate of LATS2 in ESCC tissues was 34.8% (57/164) and the low expression rate of LATS2 in these tissues was 65.2% (107/164). Downregulation of LATS2 significantly correlated with well histological grade (*p* = 0.028), lymph node metastasis (*p* = 0.003) and pTNM clinical stage (*p* = 0.005), while no significant correlations were observed with other clinicopathological parameters (Table 2). Additionally, the Kaplan-Meier survival plots revealed patients with low levels of LATS2 expression had a poorer overall survival (OS, 34 months vs. 85 months, *p*<0.001 = and progress-free survival (PFS, 20 months vs. 73 months, *p*<0.001 = than those with high LATS2 expression (Fig. 2h, i). Taken together, these results suggested that the expressions of LATS2 were bound up with the occurrence and progress of ESCC, and it may account for the development and progression of ESCC.

**Table 2:** Correlation between LATS2 expression and clinicopathological features

Patient characteristics	n	LATS2		*P*
		–	+	
Gender				0.973
Male	135	88	47	
Female	29	19	10	
Age				0.881
≤ 58	85	55	30	
> 58	79	52	27	
Tumor diameter				0.077
<5 cm	106	64	42	
≥ 5 cm	58	43	15	
Tumor location				0.603
Upper	15	9	6	
Middle	104	66	38	
Lower	45	32	13	
T stage				0.182
T1–2	69	41	28	
T3–4	95	66	29	
Lymph node metastasis				0.003
Negative	104	59	45	
Positive	60	48	12	
Histological grade				0.028
Well	31	17	14	
Middle	97	60	37	
Poor	36	30	6	
pTNM stage				0.005
I	28	14	14	
II	85	51	34	
III	51	42	9	

### Effects of LATS2 on ESCC cell growth, colony formation, migration, and invasion in vitro

**Fig. 3:**
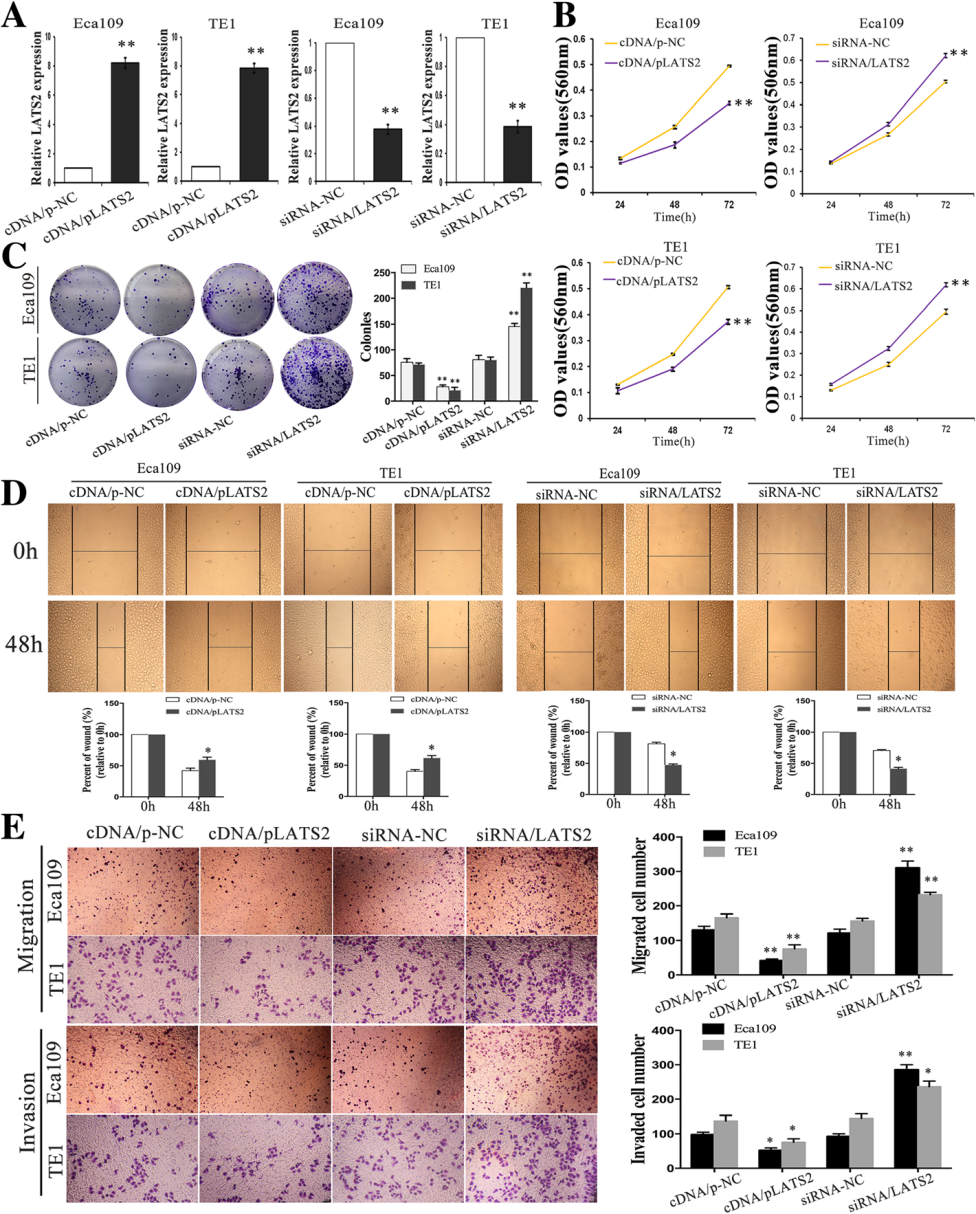
Effects of LATS2 on ESCC cell proliferation, colony formation, migration, and invasion in vitro. **a** QRT-PCR analysis of the relative expression of LATS2 in two groups of ESCC cells transfected with cDNA/pLATS2, siRNA/LATS2 and their corresponding control. **b**-**c** MTT and colony formation assays in ESCC cells after changed expressing of LATS2. **d** Wound scratch healing assay was performed to evaluate the motility of ESCC cells transfected with cDNA/pLATS2, siRNA/LATS2. Photographs were taken immediately (0 h) and at 48 h after wounding, quantification of wound closure was done. **e** Migration assay and Invasion assay were conducted to examine the invasion ability of ESCC cells. Results are expressed as the mean ± SD of three independent experiments. *: *P* < 0.05; **: *P* < 0.01

In order to investigate whether the discrepant expression of LATS2 is responsible for the tumorigenesis of ESCC, transfection of LATS2 eukaryotic expression vectors and LATS2 interference vector (named cDNA/pLATS2 and siRNA/LATS2, respectively) was performed in ESCC cell lines. After transfection of 48 h, satisfactory transfection efficiency was evaluated (Fig. 3a). Upregulation of LATS2 significantly intimidated ESCC cell viability and colony formation ability (Fig. 3b, c), while silencing of LATS2 expression stimulated cell growth compared with the control group. Then we determined whether LATS2 could meddle the migration and invasion of ESCC cells. Obviously, wound healing assay disclosed that overexpression of LATS2 constrained the migratory activity of ESCC cells, yet loss of LATS2 promoted the migratory activity (Fig. 3d). Meanwhile, Invasion assay demonstrated that enforced expression of LATS2 reduced the invasiveness of ESCC cells, and knockdown of LATS2 caused the contrary effect. Consistently, matrigel invasion assay also identified a significant augment in cell invasion after siRNA/LATS2 transfection compared with control cells (Fig. 3e). Taken together, these comments proved that LATS2 could refrain ESCC progression by inhibiting cell proliferation, invasion, and migration.

### Silencing of LATS2 reverses the effects of anti-miR-31 on phenotypes of ESCC cells

**Fig. 4:**
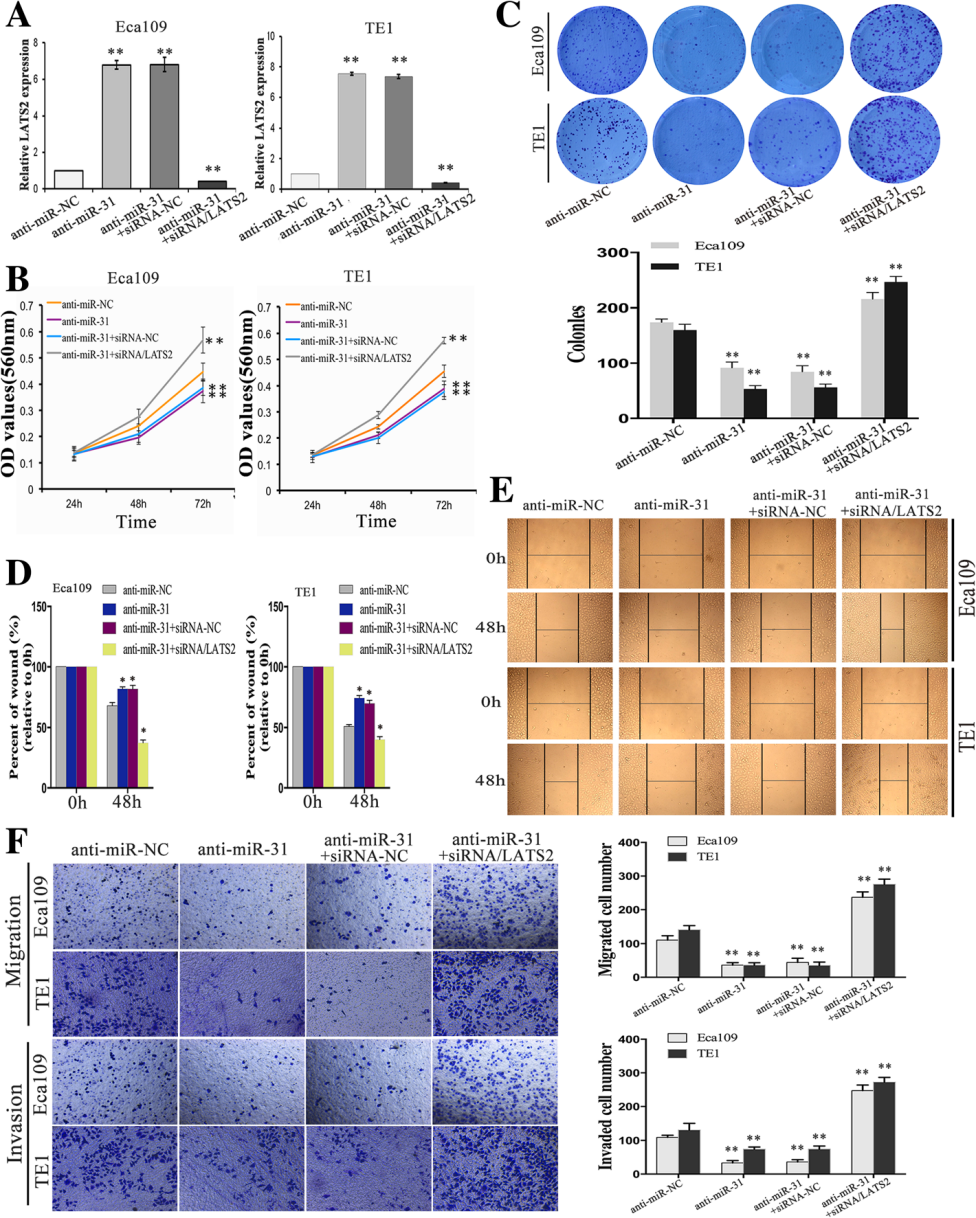
Silencing of LATS2 reverses the effects of anti-miR-31 on phenotypes of ESCC cells. **a** Quantification of LATS2 expression was achieved by qRT-PCR transfected with anti-miR-31 or co-transfected with siRNA/LATS2. **b**-**c** MTT and colony formation assays were conducted to evaluate the proliferative capacity of ESCC cells. **d**-**e**. Wound healing assays were used to assess the motility of ESCC cells transfected with anti-miR-31 alone or co-transfected with siRNA/LATS2. **f** Migration and invasion assay were conducted in ESCC cells transfected with the same type. Results are expressed as the mean ± SD of three independent experiments. *: *P* < 0.05; **: *P* < 0.01

We then determined the mechanism underlying the tumor development effect of miR-31 and decreased whether LATS2 is involved in this process. At first, anti-miR-31-expressing and siRNA/LATS2 constructs were stably co-transfected into Eca109 and TE1 cells with their relevant control, and qRT-PCR confirmed the clearly transformation of LATS2 mRNA (Fig. 4a). Next, colony formation and MTT assays presented that miR-31 downexpression both enhanced colony formation ability and cell growth rate, whereas co-transfection of anti-miR-31 and siRNA/LATS2 significantly blocked this strengthened growth effect (Fig. 4b, c). Additionally, migration and invasion analyses validated that cells co-transfected with anti-miR-31 and siRNA/LATS2 occurred increase of migratory and invasive capacities in comparison to cells only transfected with anti-miR-31 (Fig. 3d–f). These findings demonstrate that LATS2 is a functional target of miR-31 and that ectopic expression of LATS2 can reverse the tumor effect of miR-31.

### MiR-31 regulates EMT in ESCC cells via suppression of LATS2

**Fig. 5:**
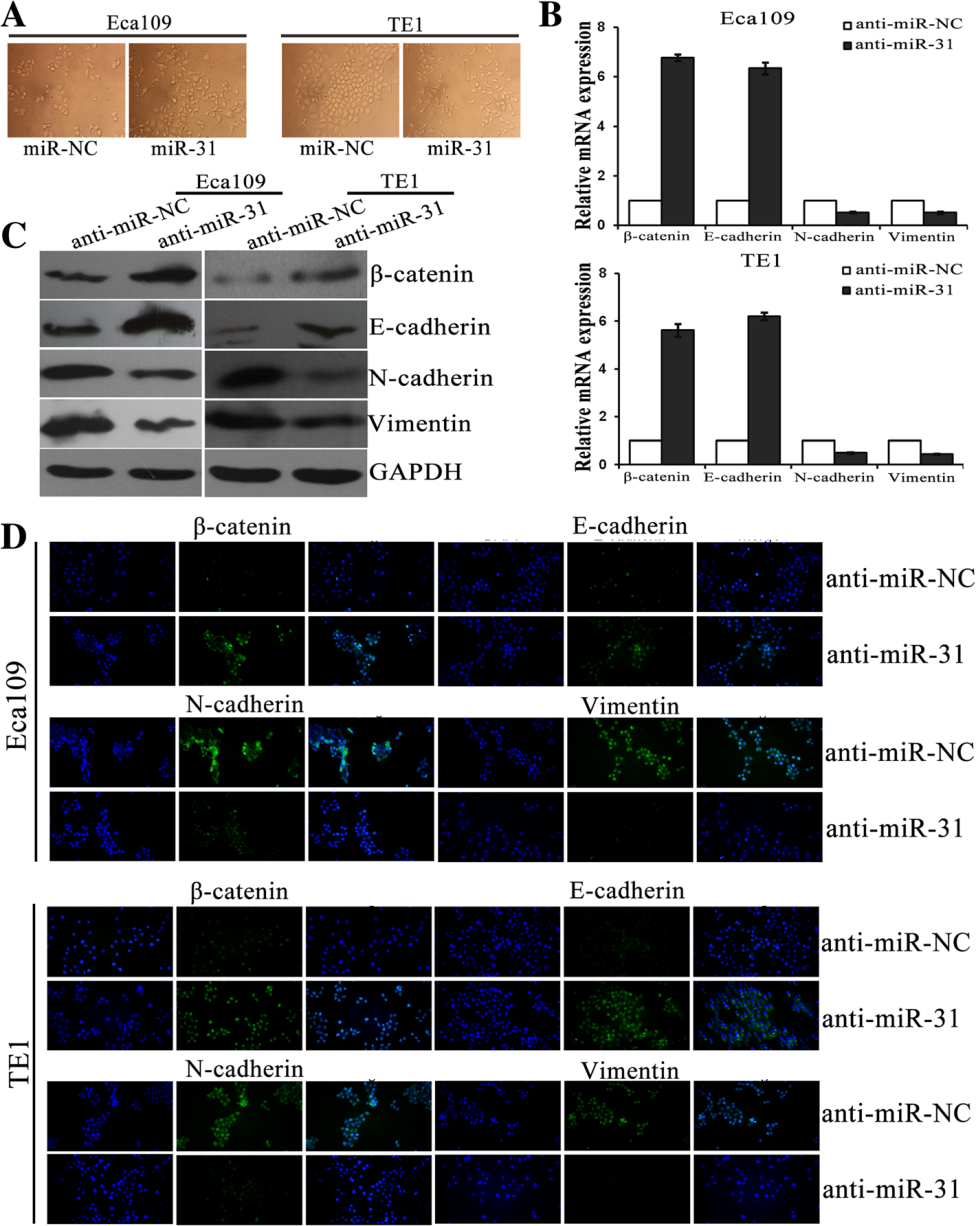
MiR-31 regulates EMT in ESCC cells. **a** Morphologies of ESCC cell lines after changing the expression of miR-31. **b**-**c** QRT-PCR and Western blotting were used to analysis the mRNA and protein levels of EMT markers in ESCC cells after transfection with anti-miR-31. **d** Immunofluorescence assay was executed the expression of EMT protein markers transfection with anti-miR-31 in ESCC cells. *: *P* < 0.05; **: *P* < 0.01

**Fig. 6:**
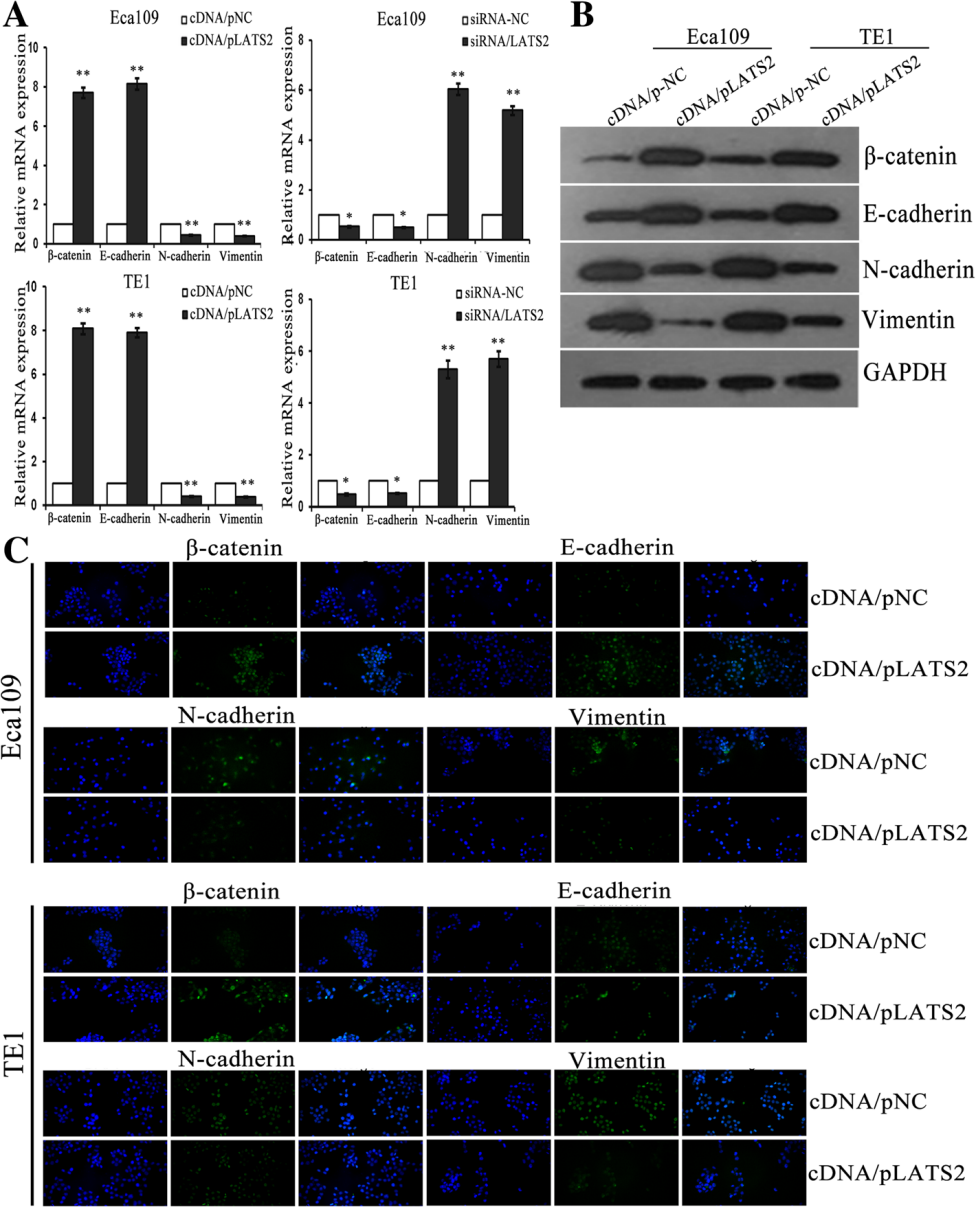
LATS2 influences EMT in ESCC cells. **a**-**b** QRT-PCR and Western blotting were used to analysis the mRNA and protein levels of EMT markers in ESCC cells after transfection with cDNA/pLATS2 and/or siRNA/LATS2. **c** Immunofluorescence assay was executed the expression of EMT protein markers transfection with cDNA/pLATS2 in ESCC cells. *: *P* < 0.05; **: *P* < 0.01

**Fig. 7:**
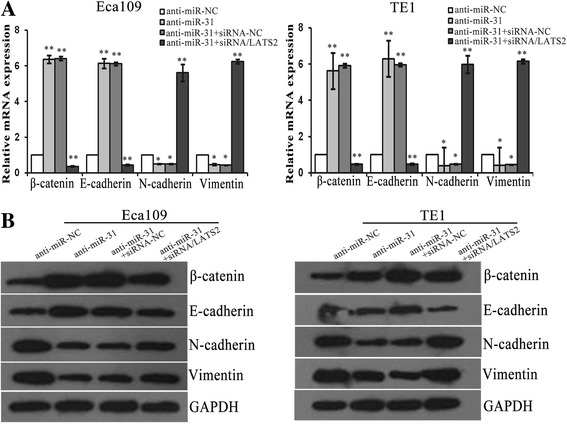
MiR-31/LATS2 interaction regulates EMT in ESCC cells. **a**-**b** QRT-PCR and Western blotting were used to analysis the mRNA and protein levels of EMT markers in ESCC cells after transfection with anti-miR-31 and co-transfection siRNA/LATS2. *: *P* < 0.05; **: *P* < 0.01

During solid tumor progression, a reactivation of epithelial tumor cells (oncogenic EMT) is regarded as one of the mechanisms that can facilitate metastatic spread [46]. Importantly, activation of EMT in epithelial cells induces a loss of cell–cell adhesions and apical-basal polarity, which is characterized by a migratory and invasive phenotype [47]. And epithelial-typed proteins usually characterized by E-cadherin is down-regulated, mesenchymal markers such as vimentin and N-cadherin are up-regulated [48]. Thus, we further determined the effects of miR-31 and LATS2 on EMT phenotypes of ESCC cells. Usually, EMT entails changes in cell morphology from epithelioid to mesenchymal. Firstly, cell morphological changes after altering the expression of miR-31 was observed. Overexpression of miR-31 in ESCC cells lines were shown to be morphologically distinct from their respective control cell lines and displayed loss of cell polarity causing a spindle-cell morphology, enlarged intercellular separation and increased formation of pseudopodia (Fig. 5a). These changes are typical of cells with a mesenchymal phenotype. Then, we detected the mRNA and protein levels of EMT markers in paired transfected cells. By means of qRT-PCR and western blotting assays, it was showed that both silencing of miR-31 and LATS2 overexpression in ESCC cells induced the expression of epithelial markers (E-cadherin and β-catenin) that was elevated by a concomitant decreased of mesenchymal markers (vimentin and N-cadherin) (Figs. 5b, c and 6a, b). Likewise, immunofluorescence assay also indicated that the expression of epithelial protein markers was significantly increased in anti-miR-31 and LATS2 overexpression transfected ESCC cells, while the expression of mesenchymal protein markers was significantly lessened (Figs. 5d and 6c). Successively, we investigated EMT-related protein expression after co-transfection to explore whether miR-31 cooperates with LATS2 to modulate EMT. Remarkably, rescue experiments demonstrated that co-transfection of anti-miR-31 and siRNA/LATS2 could reverse not only the improved expression of anti-miR-31 and epithelial markers but also the abridged expression of mesenchymal markers in Eca109 and TE1 cells induced by miR-31 upregulation (Fig. 7a, b). These results verified a vital role of miR-31/LATS2 axis in modulating EMT processing in ESCC progression.

### LATS2 overexpression leads to the reduction of TAZ signaling, which induces tumor development and correlates with prognosis in ESCC

**Fig. 8:**
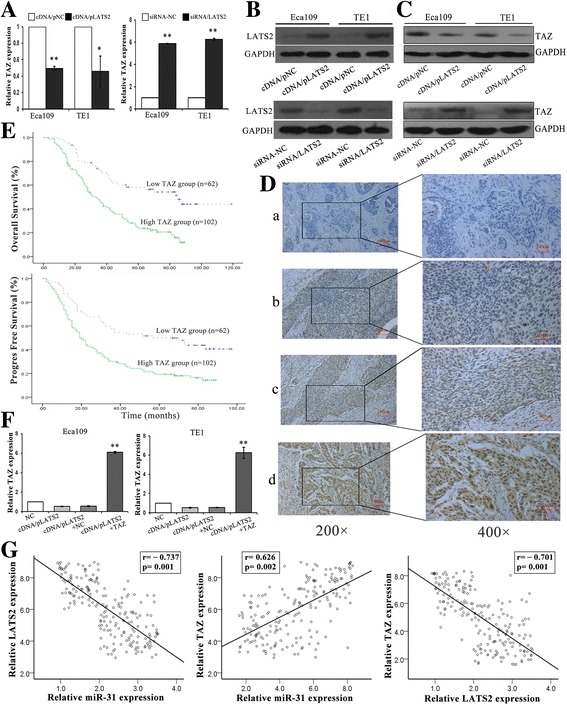
LATS2 overexpression leads to the reduction of TAZ, which induces tumor development and correlates with prognosis in ESCC. **a**-**c** QRT-PCR and western blot analysis showed that LATS2 could negatively regulate TAZ expression of ESCC cells. **b** Western blot for LATS2 antibody. **d** Immunohistochemical staining of TAZ protein in primary ESCC tissue samples (Left: ×200; Right: ×400). **e** Kaplan-Meier survival plots analysis of the association of TAZ with the OS and PFS of ESCC patients. **f** Rescue assay confirmed that overexpression of TAZ partially reverse the effects of LATS2 inhibition in ESCC cells via qRT-PCR Values are mean ± SD. **g** Analysis of correlation between miR-31 and LATS2/TAZ, LATS2 and TAZ expression levels in 164 ESCC tissues specimens. *: *P* < 0.05; **: *P* < 0.01

Several evidences supported the phosphorylation of TAZ by LATS2 with its functional inhibition [32]. In order to address whether LATS2 directly inhibited TAZ and determine the function of LATS2/TAZ in ESCC prognosis, we performed qRT-PCR and western blotting assays and observed that both TAZ mRNA and protein levels were declined and increased respectively, which transfected cDNA/LATS2 and siRNA/LATS2 (Fig. 8a–c). Then, TAZ were also analyzed by MaxVision immunohistochemical method in 164 ESCC tissues, and their correlation with clinicopathological features. TAZ was expressed in nucleus and staining intensity was scored as follows: a (blank control), b (weak staining), c (moderate staining), and d (strong staining). PBS solution was used as blank control (Fig. 8d). The high expression rate of TAZ in ESCC tissues was 62.2% (102/164) and the low expression rate of LATS2 in these tissues was 37.8% (62/164). Moreover, TAZ expression level was correlated with depth of invasion (*p* = 0.024), lymph node status (*p* = 0.025), tumor size >5 cm (*p* = 0.003), worse tumor differentiation (*p* = 0.043) and pTNM stages (*p* = 0.013) in ESCC tumor tissues (Table 3). And the association of TAZ expression with LATS2 expression in ESCC tissures was analysed. Among 102 samples with high expression of TAZ, LATS2 overexpression samples were 24 cases, the other 78 were downexpression; Meanwhile, 62 samples with low expression of TAZ, high expression of LATS2 at 33 cases, low at 29 cases. Statistical analysis revealed that TAZ expression was negatively correlated with the expression of LATS2 in ESCC tissues (*P*<0.001 = (Table 4). Overall survival (OS) and progression-free survival (PFS) were further investigated. The median OS and PFS of patients with TAZ high expression were 33 months and 19 months, lower than 85 months and 57 months of patients with low expression (*P* < 0.001) (Fig. 8e). Additionally, qRT-PCR confirmed that overexpression of TAZ partially reversed the effects of LATS2 inhibition in ESCC cells (Fig. 8f). Furthermore, in the same ESCC patients the inverse association between miR-31 and LATS2 was significant (*r* = −0.737, *p* = 0.001) and miR-31 and TAZ has a positive correlation(*r* = 0.626, *p* = 0.002) based on Pearson Correlation analysis. We also demonstrated a notable negative correlation between LATS2 and TAZ (*r* = −0.701, p = 0.001) (Fig. 8g). These data indicated that LATS2 could negatively regulate the expression of TAZ, and overexpression of TAZ partially reversed the effects of LATS2 downregulation in ESCC cells. In the same ESCC patients, miR-31 and LATS2 is anti-correlated, as well as miR-31 and TAZ is positively correlated. Moreover, the expression of TAZ was clearly correlated with metastasis and prognosis in ESCC.

**Table 3:** Correlation between TAZ expression and clinicopathological features

Patient characteristics	n	TAZ		*P*
		–	+	
Gender				0.988
Male	135	51	84	
Female	29	11	18	
Age				0.356
≤ 58	85	35	50	
> 58	79	27	52	
Tumor diameter				0.003
<5 cm	106	49	57	
≥ 5 cm	58	13	45	
Tumor location				0.415
Upper	15	8	7	
Middle	104	37	67	
Lower	45	17	28	
T stage				0.024
T1–2	69	33	36	
T3–4	95	29	66	
Lymph node metastasis				0.025
Negative	104	46	58	
Positive	60	16	44	
Histological grade				0.043
Well	31	16	15	
Middle	97	38	59	
Poor	36	8	28	
pTNM stage				0.013
I	28	17	11	
II	85	31	54	
III	51	14	37

**Table 4:** Statistical analyses revealed that TAZ expression was negatively correlated with the expression of LATS2 in ESCC tissues

TAZ	Lats2		r	*P*
	Low	High		
Low	29	33	−0.302	0.000
High	78	24		

### MiR-31/LATS2/TAZ interaction regulates tumor growth in vivo

**Fig. 9:**
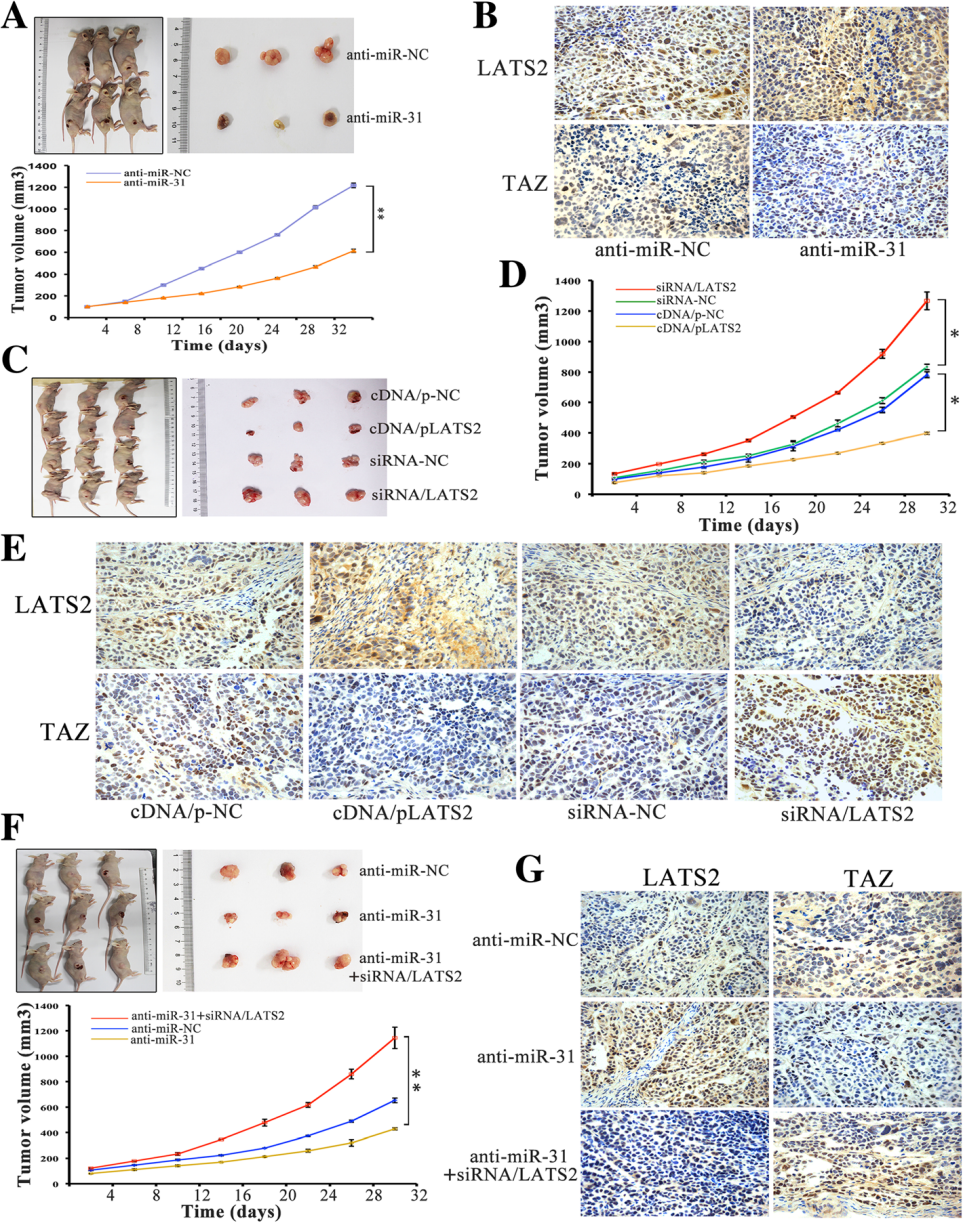
MiR-31/LATS2/TAZ interaction regulates tumor growth in vivo. **a**-**b** Effect of miR-31 on tumor growth in nude minces. Growth curves of tumors resulted from injection of Eca109 cells transfected with anti-miR-31 in nude mice. Immunostaining of LATS2 and TAZ protein stained sections of the transplanted tumors as indicated (original magnification, ×400). **c**-**e** Effect of different LATS2 expression on tumor growth in nude mice. Xenograft assay with TE1 stable cells revealed that inhibition of LATS2 improved the volume of the xenograft tumors, while restoration of LATS2 showed a significantly depressed tumor volume. **f**-**g** Loss of LATS2 significantly reversed the suppression of tumor growth induced by anti-miR-31. Immunostaining of LATS2 and TAZ protein stained sections was indicated (original magnification, ×400). *: *P* < 0.05; **: *P* < 0.01

To investigate the role of miR-31 in tumor growth in vivo by subcutaneous injection of ESCC cells transfected as described above into the flank of nude mice. Almost 8 days after inoculation, all mice developed tumors and were sacrificed after 32 days. Besides, we measured expression levels of LATS2 and TAZ protein in ESCC cells in vivo using immunohistochemistry analysis resected tumor tissue sections. As shown in Fig. 9a, loss of miR-31 expression significantly reduced the growth volume and rate of Eca109 cells-derived tumors in mice compared with anti-miR-NC cells. Clearly, staining of LATS2 protein was greatly increased in the anti-miR-31 transfected groups, whereas TAZ protein staining was diminished (Fig. 9b). Furthermore, the role of LATS2 in tumor growth was examined using xenograft mouse models and uncovered that upregulation of LATS2 attenuated the tumor growth rate and reduced tumor volume in vivo (Fig. 9c, d). And histological analysis of tumor sections and found that LATS2 upregulation and downregulation cells exhibited increased and decreased LATS2 protein staining in comparison to control groups (Fig. 9e), respectively. Additionally, restoration of siRNA/LATS2 significantly increased tumor volume (Fig. 9f). Immunohistochemistry was also performed to detect the expression of LATS2 and TAZ. Silencing of LATS2 reverses the effects of anti-miR-31 on increased LATS2, while TAZ was accordingly enhanced (Fig. 9g). Moreover, the survival analysis on 164 patients’ specimens was performed to investigate the effect of LATS2 and TAZ expression on prognosis using TCGA expression data. There were no statistical differences in the analysis results (Additional file 1: Figure S1). We are trying our best to do further explorations and analysis to acquire deeper understanding about the metastasis mechanisms of LATS2 and TAZ. These studies showed that both reduced miR-31 expression and LATS2 augment consistently led to inhibit tumor growth. These results verified a vital role of miR31/ LATS2/TAZ axis in modulating ESCC progression.

## Discussion

Altered expression of miRNAs are frequently observed in human cancers and converged to maintain distinctive characters of various processes, including ESCC. However, the mechanisms underlying their regulation and modulating carcinogenesis and progression are poorly understood. It’s speculated that miR-31 could be an oncogene in several cancers [12, 49–51] and tumor suppressor gene in several others [52–54]. MiR-31 has a specific function in different types of malignancies and processes, including cell proliferation, metastasis and EMT. Yet, little is known about the miR-31 status in patients with esophageal cancer. In this study, we reported that miR-31 acted as an oncogene in the development of ESCC by directly inhibiting LATS2 expression and ulteriorly stimulating TAZ, ultimately triggering EMT in cancer cells. Our study also demonstrated that the relationship between LATS2 and TAZ expression levels and the clinicopathological features and outcomes of ESCC patients. Moreover, we reported for the first time that miR-31 could directly silence LATS2 expression inhibiting EMT in ESCC cancer cells. Besides, we performed Pearson Correlation analysis that validated miR-31 and LATS2 is anti-correlated as well as miR-31 and TAZ is positively correlated in the same ESCC patients, a notable negative correlation between LATS2 and TAZ was also demonstrated. MiR-31/LATS2/TAZ axis could be potential novel molecular markers for predicting the risk of recurrence and prognosis of ESCC.

In previous studies reporting miR-31 to be an oncogene in ESCC [20], ectopic expression of miR-31 in ESCC and EAC cell lines leads to down regulation of SOX4, EZH2 and HDAC3, inhibiting growth, migration, and invasion of these cell lines [55]. In Zinc deficiency esophagus and tongue cancers, oncogenic miR-31 overexpression was accompanied by down-regulation of their respective tumor-suppressor targets PPP2R2A and PDCD4 [50]. Thus, it is also plausible that miR-31 expression and function in ESCC patients and cell lines. In this study, we demonstrated that miR-31 could function as an oncogene in ESCC. Declined expression of miR-31 significantly blocked ESCC cells proliferation and inhibited growth of xenograft tumors in nude mice. Besides, our study also revealed that loss of miR-31 effectively repressed cells migratory and invasive abilities.

The main challenge faced by more experiments is the mechanisms underlying miR-31 induced ESCC proliferation and metastasis. By conducting dual-luciferase reporter assay, LATS2 was identified as a direct downstream target gene of miR-31. As a member of tumor suppressors, LATS2 could play a central role in the Hippo pathway in the inhibition of cell growth and tumor suppression [56]. Recently, a considerable number of researches have grown up around the theme of miRNA/LATS2 axis involved in tumor development. It was publicized that restoration of LATS2 significantly attenuated the oncogenic effects of miR-25 [57]. Furthermore, miR-373 affected the esophageal cancer cells growth through inhibition of LATS2 expression [26]. MiR-181b was also reported to promote ovarian cancer cell growth and invasion by targeting LATS2 [40]. In addition, miR-93 enhanced angiogenesis and metastasis by targeting LATS2 [41]. Analogously, Mitamura T et al. validated that miR-31 could suppress the luciferase activity of mRNA combined with the LATS2 3′-UTR and consequently promoted the translocation of YAP1 [45]. A study mentioned that the Hippo pathway kinases LATS1/2 control activation of the transcriptional coactivators TAZ in hepatocytes and biliary epithelial cells (BECs) thereby regulating liver cell proliferation, differentiation and malignant transformation [58]. Proverbially, the activity of the LATS1/2 kinases could phosphorylate and inhibited TAZ, which was reported in different investigations [32, 38, 58]. Contrasting with these observations in other cancer types, our experiments demonstrated dramatic downregulation of LATS2 in ESCC tissues and cell lines, and a correlation between LATS2 expression levels and tumor metastasis and prognosis in ESCC patients. Improved LATS2 expression inhibited ESCC cells proliferation and metastasis. Furthermore, the inhibitory effects of anti-miR-31 on ESCC cell proliferation, migration, and invasion were reversed by restoration of downregulation LATS2 expression. Besides, LATS2 overexpression led to the reduction of TAZ signaling. Statistical analysis similarly revealed that TAZ expression was negatively correlated with the expression of LATS2 in ESCC tissues. And we evaluated the relationship between TAZ expression levels and the clinicopathological features, and outcomes of ESCC patients. Survival analysis showed that low Lats2 expression associated with better prognosis, but high expression of TAZ presaged shorter survival period. Any links of Hippo pathway caused by out of control, LATS2 and/or TAZ expression might get out of control, which would lead to unrestricted cell growth and movement ability. The generalizability of these results was subjected to certain limitations, deserving further investigation in a larger patient cohort. We considered that our conclusions challenge the current discussion of the role of TAZ in tumor progression, further explorations will be made on the mechanisms of TAZ and get a deeper understanding of Hippo pathway in ESCC progression.

EMT, the key process driving invasiveness and metastasis, is originally defined as a morphological conversion during embryogenesis. The concept of EMT is characterized by loss of the epithelial marker, increased expression of the mesenchymal marker, and enhanced migratory and invasive behaviors [59]. And morphological features of EMT have been mostly described in human cancers of epithelial origin [60]. EMT plays crucial roles during tumor metastasis and is one of the major molecular mechanisms through which invasion and metastasis are promoted during the ESCC oncogenic process [61, 62]. Here, we further measured the expression of EMT regulatory proteins in ESCC cells. Chiefly, levels of the epithelial markers β-catenin and E-cadherin were dramatically increased in anti-miR-31 and cDNA/pLATS2 transfected cells. Meanwhile, levels of the mesenchymal markers N-cadherin and vimentin were diminished in both groups of transfected cells. Consistently, immunofluorescence assay showed the same trend. Thus, we identified miR-31-mediated LATS2 signaling pathways to be involved in cancer EMT process, which is a pivotal step for ESCC metastasis.

## Conclusions

In conclusion, we herein demonstrated that miR-31 could exhibit oncogenic roles and promote ESCC tumorigenesis by inhibiting the expression of LATS2, which negatively regulated TAZ. Moreover, dysregulation of miR-31/LATS2/TAZ axis might be a novel molecular mechanism involved in the development and progression of ESCC.

## Additional file

Additional file 1: Figure S1. Survival analysis was displayed using TCGA expression data. There were no statistical differences in the analysis results. A. Survival analysis on 164 patients’ specimens was performed to investigate the effect of LATS2 and TAZ expression on prognosis using TCGA expression data. B. Survival analysis on patients with metastasis from 164 specimens was performed to investigate the effect of LATS2 and TAZ expression on metastasis using TCGA expression data. (TIFF 179 kb)

## Abbreviations

3′-UTR: 3′-untranslated regions
EA: esophageal adenocarcinoma
ESCC: esophageal squamous cell carcinoma
GAPDH: glyceraldehyde-3-phosphate dehydrogenase
IHC: immunohistochemistry
LATS2: Large tumor suppressor homolog 2
OS: overall survival
PBS: phosphate-buffered saline
PFS: progress-free survival
qRT-PCR: real-time quantitative reverse-transcription polymerase chain reaction
siRNA: small interfering RNA
