# Supplementary figures and images for: Downregulation of MiR-31 stimulates expression of LATS2 via the hippo pathway and promotes epithelial-mesenchymal transition in esophageal squamous cell carcinoma
Source: J Exp Clin Cancer Res. 2017 Nov 16;36:161. doi: 10.1186/s13046-017-0622-1 (PMC5689139; doi:10.1186/s13046-017-0622-1)

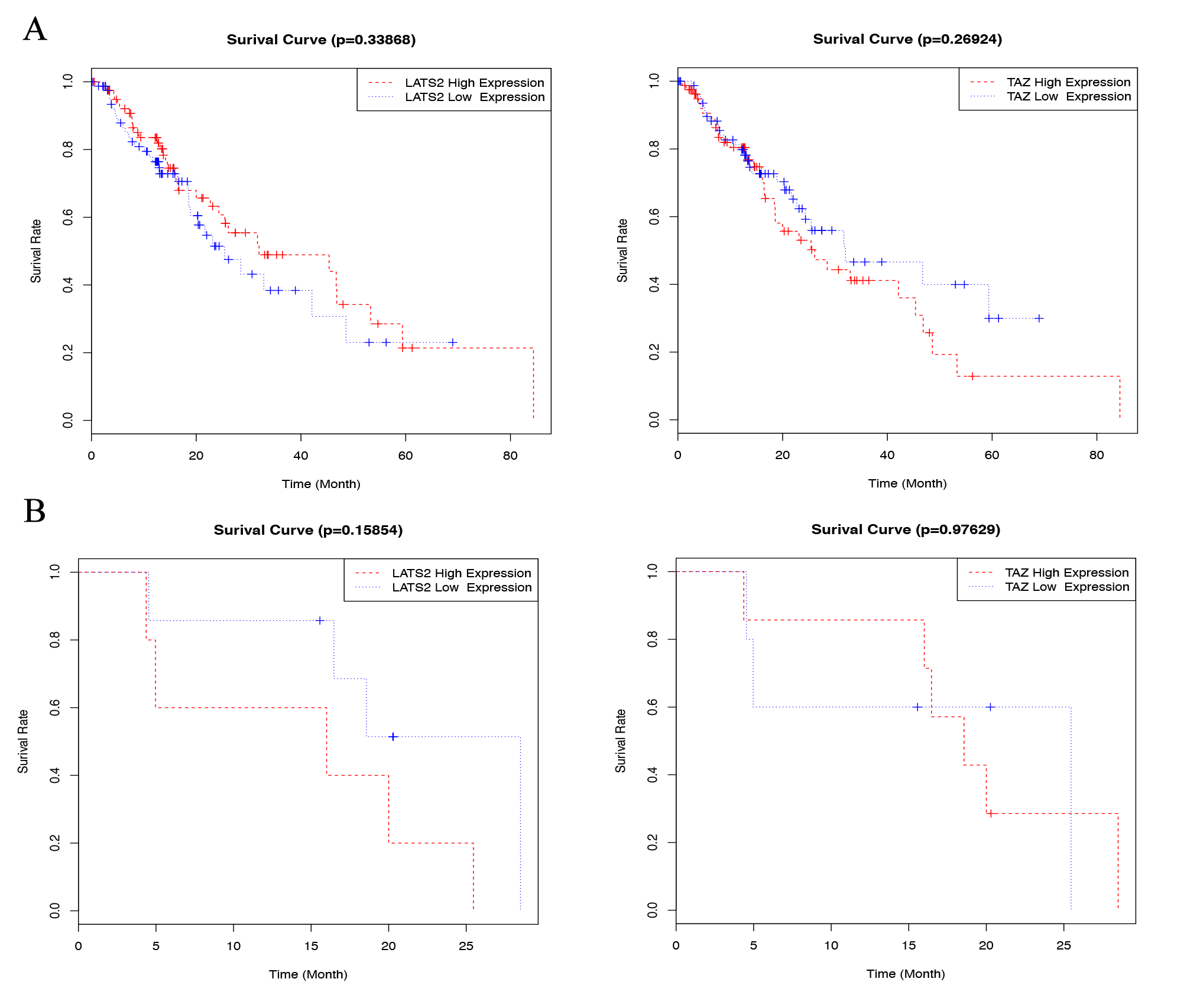

Supplement: Additional file 1: Figure S1. — Survival analysis was displayed using TCGA expression data. There were no statistical differences in the analysis results. A. Survival analysis on 164 patients’ specimens was performed to investigate the effect of LATS2 and TAZ expression on prognosis using TCGA expression data. B. Survival analysis on patients with metastasis from 164 specimens was performed to investigate the effect of LATS2 and TAZ expression on metastasis using TCGA expression data. (TIFF 179 kb) [file 13046_2017_622_MOESM1_ESM.tif]
